# Comparison between the Express Implant and Transscleral Diode Laser in Neovascular Glaucoma

**DOI:** 10.1155/2020/3781249

**Published:** 2020-05-17

**Authors:** Faried Mohammed Wagdy, Adel Galal Zaky

**Affiliations:** Ophthalmology Department, Faculty of Medicine, Menoufia University, Shebeen El-Kom, Egypt

## Abstract

**Purpose:**

To compare the outcomes of Ex-PRESS glaucoma filtration device and transscleral cyclophotocoagulation (TSCP) in the management of neovascular glaucoma (NVG). *Patients and Methods*. A total of 30 eyes (12 express shunts and 18 TSCP) of 28 patients were included. The eyes had NVG with intraocular pressure (IOP) more than 21 mmHg of the maximally tolerated medication treatment after previous panretinal photocoagulation and antivascular endothelial growth factor (anti-VEGF) injection, with no previous history of a cyclodestruction procedure or glaucoma surgery, were randomized either for Ex-PRESS glaucoma filtration device or TSCP. The patients were followed up weekly for the first month and then monthly for 12 months as regard to the IOP, number of topical antiglaucoma drugs required, visual outcome, and postoperative complications.

**Results:**

IOP was successfully controlled with both techniques in 83.3% of the eyes. Both techniques had fewer complications and required fewer subsequent procedures.

**Conclusion:**

Both the Ex-PRESS glaucoma filtration device and TSCP might constitute safe and alternative therapeutic tools for patients with NVG. However, TSCP is an easier procedure, less time consuming, and does not require a learning curve.

## 1. Introduction

Neovascular glaucoma (NVG) is a refractory glaucoma that often results in loss of vision despite aggressive management. Numerous interferences and medications have been used for the control of raised intraocular pressure (IOP) in NVG, but no definite therapeutic tool has been recognized as the most effective armamentarium [1]. Surgery is usually considered if medication and laser treatment fail to control IOP [2]. Trabeculectomy is the most common type of glaucoma filtration surgery and is considered the mainstay of glaucoma surgeries [3, 4]. However, trabeculectomy is still associated with some postoperative complications, including hyphema, bleb leak, hypotony, choroidal detachment, bleb failure, blebitis, and endophthalmitis [5].

Glaucoma drainage devices are commonly and effectively used in the surgical treatment of NVG [6]. The Ex-PRESS glaucoma filtration device (Alcon Laboratories, Fort Worth, TX) is a nonvalved stainless steel implant that drains aqueous humor from the anterior chamber into the subconjunctival space and has been used as an alternative to trabeculectomy [7]. The Ex-PRESS glaucoma filtration device has the advantage of being less traumatic than traditional trabeculectomy as it does not require a sclerectomy or peripheral iridectomy [8].

Diode laser transscleral cyclophotocoagulation (TSCP) is also commonly used in NVG, and high rates of successful control of IOP have been reported. TSCP also has fewer complications and is less aggressive compared to cyclocryotherapy [9–12].

This study aims to compare the outcomes of the Ex-PRESS glaucoma filtration device and transscleral cyclophotocoagulation (TSCP) in the management of NVG.

## 2. Patients and Methods

This prospective case series study was performed at Menoufia University Hospital and the Eye Vision Specialized Center from September 2017 to March 2019. Patients with neovascular glaucoma who met the eligibility criteria were included. Ethical approval was received from the Ethical Committee of Menoufia Faculty of Medicine, and the Declaration of Helsinki was followed.

We included 28 patients aged between 35 and 55 years old with a clinical diagnosis of NVG and IOP greater than 21 mmHg despite maximal medical treatment. Eyes with no light perception (NLP) or a previous history of glaucoma surgery or cyclodestruction were excluded.

Patients were randomized using a computer-generated random number table. All the patients had received multiple antivascular endothelial growth factor (anti-VEGF) injections as a therapeutic tool for the primary disease. Preoperative panretinal photocoagulation (PRP) was performed whenever there was good fundus visualization, or postoperatively when the fundus visualization improved.

Patients were randomized for the Ex-PRESS glaucoma filtration device; 12 eyes of 12 patients (group A) underwent the procedure in the operating room under either regional anesthesia (peribulbar block with 2% lidocaine) or general anesthesia.

The surgical technique used for the Ex-PRESS glaucoma filtration device was similar to that used for trabeculectomy without sclerectomy or peripheral iridectomy. Briefly, the procedure involved conjunctival peritomy with fornix-based conjunctival flap creation, light diathermy, and a large Weck‐cell sponge soaked in a 0.2 mg/ml solution of MMC was placed for 2 minutes, followed by copious irrigation with a balanced salt solution. A scleral triangular flap was placed forward of the clear cornea to allow exposure of the scleral spur, and a pilot hole was created using a sapphire blade (Alcon laboratories, USA). The Ex-PRESS glaucoma filtration device was then implanted with its tip in the anterior chamber, midway between the iris periphery and the cornea. Finally, the scleral flap was closed using monofilament 10/0, and the conjunctiva was closed using vicryl 8/0.

Postoperatively, prednisolone acetate 1% and moxifloxacin 0.5% eye drops were administered every 2 hours for at least 4 weeks and then gradually withdrawn according to the clinical response. All previous glaucoma medications were stopped postoperatively and resumed according to IOP measurements.

TSCP subjects (18 eyes of 16 patients (group B)) received treatment under regional anesthesia (peribulbar block with 2% lidocaine). A diode laser with a wavelength of 810 nm was delivered using the G-probe (OcuLight SLx; IRIS Medical Instruments, USA) for 270° around the limbus, with eight shots per quadrant (24 shots in total). The duration was set as 2 seconds, and the initial power was set as 1.75 W. A “pop”-titrated protocol was used to adjust laser power such that it was decreased after every two consecutive audible shots (“pop”) and increased after every two consecutive silent shots. Postoperatively, prednisolone acetate 1% was administered every 3 hours and atropine 1% eye drops was prescribed twice daily and then gradually withdrawn according to clinical response [13]. All previous glaucoma medications were modified according to the IOP measurements during follow-up visits. The laser procedure was repeated if necessary, leaving at least 4 weeks between every session, until either the IOP was ≤ 21 mmHg or a total of five sessions had been reached.

Both groups were followed up weekly for the first month and then monthly for at least 12 months with regards to their IOP, number of topical antiglaucoma drugs required, best-corrected visual acuity (BCVA), and postoperative complications.

## 3. Outcome Analysis

### 3.1. IOP and Number of Glaucoma Medications

IOP was measured with a Goldmann applanation tonometer at baseline and subsequent follow-ups. Complete success in IOP was considered as IOP < 22 mmHg without treatment, a qualified success in IOP was considered as IOP < 22 mmHg with medical treatment, and failure in IOP control was considered if the IOP was ≥22 mmHg after surgery.

### 3.2. Visual Acuity

The BCVA (in decimals) of each eye at the final visit was compared to the preoperative BCVA. Changes in the final BCVA were categorized as “worsened,” “stable,” or “improved” compared to the preoperative BCVA. A change of one line of Snellen visual acuity or less was defined as stable, whereas greater changes were defined as worsened or improved accordingly. For BCVA not better than count fingers (CF), this was defined as improved if it changed from light perception only (LP) to hand movement (HM) or better, or from HM to CF or better. The reverse applied for worsened visual acuity.

### 3.3. Complications

All intraoperative and postoperative complications were recorded.

### 3.4. Statistical Analyses

The database was maintained and managed using Microsoft Excel 2016 (Microsoft Corporation, Redmond, WA, USA). All statistical analyses were performed using SPSS software version 16.0 (SPSS Inc., Chicago, IL, USA). For descriptive statistics, continuous variables were expressed as mean ± SD (range), whereas categorical variables were presented as frequencies with percentages. The chi-square test or Fisher's exact test was used to compare the proportions of subjects with visual stability or success. A *P* value of ≤0.05 was considered statistically significant.

## 4. Results

**Figure 1:**
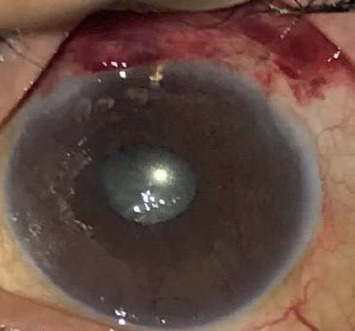
Express shunt after 1 week.

**Figure 2:**
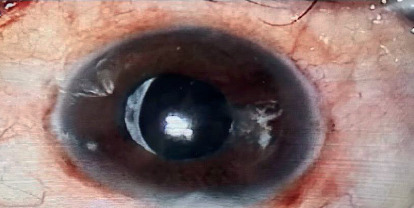
Appearance of the eye after contact transscleral diode laser cyclophotocoagulation.

A total of 30 eyes of 28 patients with NVG were included in this study. The Ex-PRESS glaucoma filtration device (Figure 1) was used in 12 eyes, and TSCP was performed in the remaining 18 eyes (Figure 2). There was no significant difference in age and sex between the patients. The demographic data and baseline clinical findings are presented in Table 1.

The main cause of NVG in our cases was proliferative diabetic retinopathy, followed by central retinal vein occlusion. Preoperative panretinal photocoagulation (PRP) was performed in 20 eyes (66.7%). For the remaining 10 eyes, 3 underwent posterior vitrectomy with endolaser PRP, while poor fundus examination due to the presence of a steamy cornea or dense cataract prevented preoperative PRP in 7 eyes and was performed later. A total of 16 eyes (53.3%) had previous cataract surgery (Table 1).

Intraocular pressure was successfully lowered in 25 eyes (83.3% of all eyes in the study); the preoperative IOP was 24–42 mmHg in all patients of both groups. There was a highly significant lowering of IOP in the first postoperative week, after 1 month, and after 1 year (*P*=0.001). A greater reduction was observed in group A, where the preoperative IOP was 28.2 ± 2.6 and declined to 15.36 ± 1.6 after 1 year (*P*=0.001); less reduction was reported in group B, where the preoperative IOP was 27.6 ± 4 and decreased to 15.44 ± 1.66 (*P*=0.001) (Table 2). However, the difference between both groups in postoperative IOP was not statistically significant.

Complete success in lowering IOP was observed in 6 eyes in group A (50%) and 8 eyes in group B (44.44%). Qualified success was observed when there was a need for postoperative antiglaucoma drugs and was observed in 5 eyes of group A (41.66%) and 7 eyes of group B (38.88%). Failure occurred in 1 eye of group A (8.33%) and 3 eyes of group B (16.66%).

Of the 18 eyes that underwent the TSCP procedure, 3 received DCPC application twice and two received TSCP three times during the eligible study period (1 year).

The number of required antiglaucoma eye drops postoperatively was reduced in both groups without any statistical significant difference (Table 3).

The postoperative VA ranged between 1/60 (one meter only) to 0.7 (by Snellen E-chart) in both groups. There was no significant postoperative change over the follow-up period that reflected stability in VA (Table 4).

With regards to the final BCVA in group A (*n* = 12), 1 had worsened, 8 remained stable, and 2 improved compared to baseline. For group B (*n* = 18), 2 had worsened, 15 remained stable, and 1 had improved BCVA. The difference in BCVA changes between the two groups was not statistically significant (*P*=0.48) (Table 5).

With regards to postoperative complications (Table 6), only a few complications were encountered in 1 eye with hyphema in each group; this resolved spontaneously without the need for surgical intervention. In group A, one eye (8.33%) had increased IOP despite the use of maximal therapy; this later required intravitreal injection of ranibizumab (0.5 mg/0.05 ml) followed by posterior vitrectomy and endolaser PRP to relieve extramacular tractional retinal detachment. In a total of three eyes in group B (16.66%), two required vitrectomy surgery and the remaining eye that had 3 previous sessions of TSCP required another session of TSCP, although this was not recorded in the study as it was performed after 1 year. Ocular hypotony was reported in 5 eyes (3 eyes in group A and 2 eyes in group B) and improved within days.

## 5. Discussion

NVG is still considered one of the most refractory and aggressive types of glaucoma. Its abnormal fibrovascular tissue growth on the iris and trabecular meshwork may lead to difficulties in decreasing IOP [13].

Glaucoma drainage devices have been advocated for primary surgical treatment of NVG since their success is thought to be less dependent on the control of intraocular inflammation and the failure of a filtering bleb [15]. The Ex-PRESS is an FDA-approved mini glaucoma implant that has been developed to simplify anterior guarded filtering surgeries, making them faster, safer, and easier [16].

In our study, we compared the success rates of the Ex-PRESS glaucoma filtration device and TSCP in patients with NVG owing to diabetic retinopathy or retinal vein occlusion. Both the Ex-PRESS glaucoma filtration device and TSCP achieved a markedly reduced IOP from a preoperative IOP of 28.2 ± 2.6 mmHg to an IOP of 15.36 ± 1.6 mmHg at the last visit in the Ex-PRESS glaucoma filtration device group (43.6% IOP reduction). In the TCPC group, the IOP was reduced from 27.6 ± 4 mmHg preoperatively to 15.44 ± 1.66 mmHg at the last visit (44.1% IOP reduction). We found that the IOP was lower in the Express implant group than the TSCP group. However, the difference between the two groups was not statistically significant.

Yu et al. [15] reported that three of four NVG patients (75.0%) who received the primary Ex-PRESS glaucoma filtration device had a postoperative IOP under 21 mmHg without any antiglaucoma medication control at the last follow-up. However, it was also shown that 3 of the 4 patients received shunt reposition due to failed blebs or recurrent NVG. In the current study, none of the patients required shunt repositioning.

Nardi et al. stated that while it is difficult to compare different reports due to a lack of uniformity in study design and because of differing success rate thresholds, there is a general consensus that the Ex-PRESS glaucoma filtration device is a safe and effective procedure for reducing IOP. The lowering effect is comparable to that of traditional trabeculectomy, and success rates are very similar using 18 mmHg as the IOP cutoff [16].

The high cost, availability of Express implant, and learning curve of the procedure play a role in selecting this option for NVG patients.

Yildirim et al. [12] reported the results of long term study of diode laser cyclophotocoagulation and the Ahmed glaucoma valve implant in NVG and showed that there was no significant difference in the success rate between both groups. However, DCPC is a less time consuming and easier method that does not require a learning curve for lowering IOP in patients with NVG. Our results match with those of Choy et al. [13] who reported that both TSCP and AGV were equally effective in reducing IOP and glaucoma medications in NVG with no previous glaucoma surgery or cyclodestruction.

With regards to the efficacy of TSCP, this study was in agreement with that of Dewundara et al. [17] who reported that the mean pretreatment IOP was 34.6 ± 10.1 mmHg for OAG, 37.6 ± 10.1 mmHg for ACG, and 38.9 ± 12.3 mmHg for NVG (*P* > 0.05). At 24 months follow-up, the mean posttreatment IOP was significantly reduced across all types of glaucoma. Furthermore, the mean posttreatment IOP at 24 months was 18.1 ± 8.8 mmHg for OAG, 25.0 ± 12.3 mmHg for ACG, and 22.4 ± 9.0 mmHg for NVG (*P* > 0.05). There was no statistically significant difference in the IOP-lowering effect of TSCP across the different types of glaucoma and at all follow-up time points. At 15 months of follow-up, a success of 39% was noted for eyes with OAG, 53% for ACG, and 17% for NVG. Of the ten eyes that required additional incisional surgery, 60% had NVG (*P* < 0.05) [17].

Several cyclophotocoagulation procedures are available for the treatment of NVG, including long-duration burn TSCP, micropulse TSCP, [18, 19] and endoscopic cyclophotocoagulation [20]; all of which aim to decrease postoperative inflammation and complications. However, more studies are needed to detect which of the above procedures is the most suitable for the treatment of NVG considering their availability and high cost which may limit wide application.

Serious complications, such as loss of light perception, blind painful eye, or atrophia bulbi, have been reported for both techniques [21]; however, none of these complications were reported in the current study.

Previous studies have reported increased levels of VEGF in the aqueous humor and Tenon tissue of patients who had unsuccessful glaucoma surgeries compared to those with successful surgeries and those without glaucoma. A certain correlation was suggested to exist between VEGF levels and the outcome of glaucoma surgery, and the potential benefit of anti-VEGF therapy was then considered for improving the success rate of glaucoma surgery [22]. Although this was beyond the scope of the current study, we emphasize that all eyes in both groups received multiple intravitreal injections as an armamentarium for the primary disease treatment plan and not as a preliminary step before interference as followed in trabeculectomy.

Both the Ex-PRESS glaucoma filtration device and TSCP are good armamentarium for controlling IOP in NVG eyes that failed to respond adequately to panretinal laser photocoagulation and topical antiglaucoma drugs. In addition, both procedures may be considered as a first effective interventional choice for lowering IOP instead of trabeculectomy surgery that carries hazards of fibrosed and failed blebs.

Limitation of our case series study includes the relatively small sample size (related to the scarcity of NVG and the long-term follow-up and high cost of the Express implant) and the lack of analysis of the combined effect of anti-VEGF with both procedures. Finally, the follow-up period was only 1 year and should be extended to assess the long-term stability of IOP.

## 6. Conclusion

Both the primary Ex-PRESS glaucoma filtration device and TSCP might constitute safe and alternative therapeutic tools for patients with NVG. However, TSCP is an easier procedure that is less time consuming and does not require a learning curve.

## Figures and Tables

**Table 1:** Demographic data and baseline clinical findings.

	Groups				*P* value
	Group A (*n* = 12 eyes) (12 patients)		Group B (*n* = 18 eyes) (16 patients)		
	No.	%	No.	%	
Age (mean ± SD)	48.66 ± 4.81		46.25 ± 5.49		0.926
Gender					0.508
Male	6	50.0	6	62.5	
Female	6	50.0	10	37.5	
Preoperative medications					0.711
Topical (beta-blocker + CAI)	5	41.7	6	33.3	
Topical (beta-blocker + CAI + brimonidine)	7	58.3	12	66.7	
Preoperative BCVA					
1/60–6/60	8	58.3	11	61.1	1.0
Better than 6/60	4	41.7	7	39.9	
Grading of NVI (Teich and Walsh, 1981) [14]					
0 no NVI	—	—	—	—	
1 < 2 quadrants of NV at the iris pupillary zone	—	—	—	—	
2 > 2 quadrants of NV at the iris pupillary zone	7	58.3	11	61.1	1.0
3 Grade 2 + <3 quadrants of NV at the iris ciliary zone and/or ectropion uveae	5	41.7	7	39.9	
4 > 3 quadrants of NV at the iris ciliary zone and/or ectropion uveae					
Causes of NVG					
PDR	8	66.7	11	61.1	1.0
CRVO	4	33.3	7	39.9	
Pre-PRP	8	66.7	12	66.7	1.0
Previous cataract surgery	7	58.3	9	50.0	0.654

**Table 2:** Difference in IOP control between the two groups.

IOP	Group A (*n* = 12 eyes)	Group B (*n* = 18 eyes)	Student's *t*-test	*P* value
Preoperative	28.2 ± 2.6	27.6 ± 4	0.629	0.532
After 1week	14.04 ± 2.96	15.44 ± 2.4	1.835	0.073
Paired *t*-test	20.605	13.690		
*P* value	0.001^*∗∗*^	0.001^*∗∗*^		
After 1 month	14.48 ± 2.43	15.44 ± 2.7	1.318	0.194
Paired *t*-test	21.737	12.527		
*P* value	0.001^*∗∗*^	0.001^*∗∗*^		
After 12 months	15.36 ± 1.6	15.44 ± 1.66	0.175	0.862
Paired *t*-test	25.072	13.837		
*P* value	0.001^*∗∗*^	0.001^*∗∗*^		

**Table 3:** Comparison of postoperative antiglaucoma medications between the two groups.

Postoperative antiglaucoma medications	Group A (*n* = 12 eyes)		Group B (*n* = 18 eyes)		*X* ^2^	*P* value
	No.	%	No.	%		
Beta-blocker + CAI + PGA	2	16.66%	3	16.66%	0.18	0.91
Beta-blocker + CAI	2	16.66%	4	22.22%		
Beta-blocker	2	16.66%	3	16.66%		

CAI, carbonic anhydrase inhibitor; PGA, prostaglandin analogue.

**Table 4:** Comparison between the two groups as regarding postoperative BCVA.

BCVA	Group A (*n* = 12 eyes)	Group B (*n* = 18 eyes)	*T*-test	*P* value
Preoperative	0.376 ± 0.153	0.164 ± 0.143	5.156	0.001^*∗∗*^
After 1week	0.372 ± 0.157	0.133 ± 0.212	4.616	0.001^*∗∗*^
*T*-test	1	0.629		
*P* value	0.327	0.536		
After 1 month	0.372 ± 0.156	0.148 ± 0.243	9.326	0.001^*∗∗*^
*T*-test	1	0.649		
*P* value	0.327	0.543		
After 12 months	0.375 ± 0.153	0.151 ± 0.199	8.760	0.001^*∗∗*^
*T*-test	—	0.668		
*P* value	—	0.614		

**Table 5:** BCVA changes in the two study groups.

BCVA changes *n* (%)	Group A (*n* = 12 eyes)	Group B (*n* = 18 eyes)	*P* value
Worsened	1 (8.33%)	2 (11.11%)	0.48
Stable	9 (75%)	15 (83.33%)	
Improved	2 (16.66%)	1 (5.55%)	

**Table 6:** Comparison of postoperative complications between the two groups.

Complications	Group A (*n* = 12)		Group B (*n* = 18)		*X* ^2^	*P* value
	No.	%	No.	%		
Hyphema	1	8.3%	1	5.5%	0.23	0.86
Increase IOP	2	16.66%	2	11.11%		
Hypotony	3	25	2	11.11%		

## Data Availability

The data used to support the findings of this study are included in the article.
